# Management of liver hydatid cyst: A case report

**DOI:** 10.1002/ccr3.7964

**Published:** 2023-09-25

**Authors:** Banwari Lal Bairwa, Ayush Anand

**Affiliations:** ^1^ Department of General and Minimal Access Surgery MP Birla Hospital and Research Center Chittorgarh India; ^2^ BP Koirala Institute of Health Sciences Dharan Nepal

**Keywords:** case report, echinococcosis, hydatid disease, management, surgery

## Abstract

Hydatid disease, if not properly managed, can lead to mortality. Open surgery is preferred and can be a curative approach for multiple and large liver hydatid cysts. Also, regular follow‐up is required to detect recurrence.

## Graphical abstract

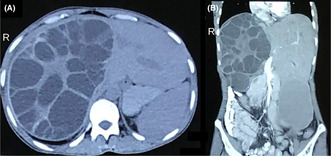

Cystic echinococcosis, also known as hydatid disease, is a zoonotic disease and leads to significant mortality if not adequately managed, particularly in Asia.^1^ Patients usually present with upper abdominal discomfort, anorexia, and weight loss.^1^, ^2^, ^3^ Herein, we report the case of a 33‐year‐old Asian female who presented with right upper abdomen swelling and pain for the last 6 months. The swelling gradually increased in size over the previous few months. The physical examination revealed significant palpable hepatomegaly 10 cm below the right subcostal margin. Her initial blood investigations revealed leukocytosis and echinococcal IgG ELISA was positive. The liver function test revealed hypoproteinemia and hypoalbuminemia. This may suggest an infected cyst seen in one fifth of patients with liver hydatid disease.^4^ The diagnosis of hydatid disease can be made based on clinical evaluation and ultrasonography of the abdomen. However, ultrasonography is less sensitive in detecting small cysts and compression effects due to enlarged liver.^1^ A computed tomography (CT) scan of the abdomen and pelvis should be used for definitive diagnosis.^2^, ^3^, ^5^ CT scan is more sensitive to detect calcification, small cysts, internal septa, daughter vesicles, and hepatomegaly's compression effects.^6^ Daughter cysts appear hypoattenuating with a honeycomb appearance and multiple internal septations representing walls of daughter cysts, reflecting Type II hydatid disease.^6^ A total calcification reflects the dead cysts and is hyperattenuating on a CT scan.^5^, ^6^ We did an urgent computed tomography scan of the abdomen and pelvis (Figure 1). It showed enlarged liver (19 cm), a well‐defined cystic lesion with internal septations, and daughter cysts in the right lobe of the liver. Additionally, compensatory hypertrophy of the left lobe of the liver, relatively higher right hemidiaphragm, and upward displacement of the liver were reported. We made a provisional diagnosis of hydatid liver disease based on the clinical evaluation. The mainstay of management in patients with large cysts is surgery.^1^, ^2^ The choice of surgery can be an open or laparoscopic approach.^1^, ^2^, ^7^, ^8^ Any of the two approaches can be used, as data regarding the superiority of one approach over the other is inconclusive.^8^ We started tablet albendazole 3 weeks before surgery and continued until 3 months after. We did an exploratory laparotomy in this case (Figure 2). The abdominal cavity was opened by Kocher incision. A surgical mop soaked in scolicidal agent (10% Povidine–Iodine) was placed around the liver cyst to prevent intraabdominal dissemination in case of spillage. 20% hypertonic saline was injected into the cystic cavity for 15 min, then opened. On exploration, the right lobe of the liver was grossly enlarged, and multiple daughter hydatid cysts occupied the whole right lobe. Approximately 300 cysts were removed from the liver (Figure 2), and partial pericystectomy was done. The remaining cavity was inspected for bile leakage, and a drainage tube was placed in the remaining cavity. The postoperative period was uneventful; the drain was removed on fourth postoperative day, and the patient was discharged on the fifth day. Interestingly, the patient had postoperative eosinophilia. In the surgical approach, there is a risk of recurrence.^1^ Hence, the patient should be kept on regular follow‐up. Our patient was under follow‐up until 3 months after the surgery and was doing well. Our case showed that open surgery could be a curative approach for large hydatid liver cysts. Additionally, regular follow‐up is required to detect recurrence.

**FIGURE 1:**
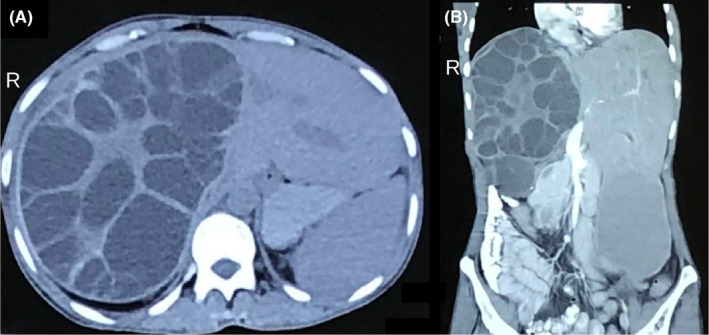
Axial (A) and coronal view (B) of computed tomography scan of the abdomen showing enlarged liver and cystic lesion with internal septations in liver.

**FIGURE 2:**
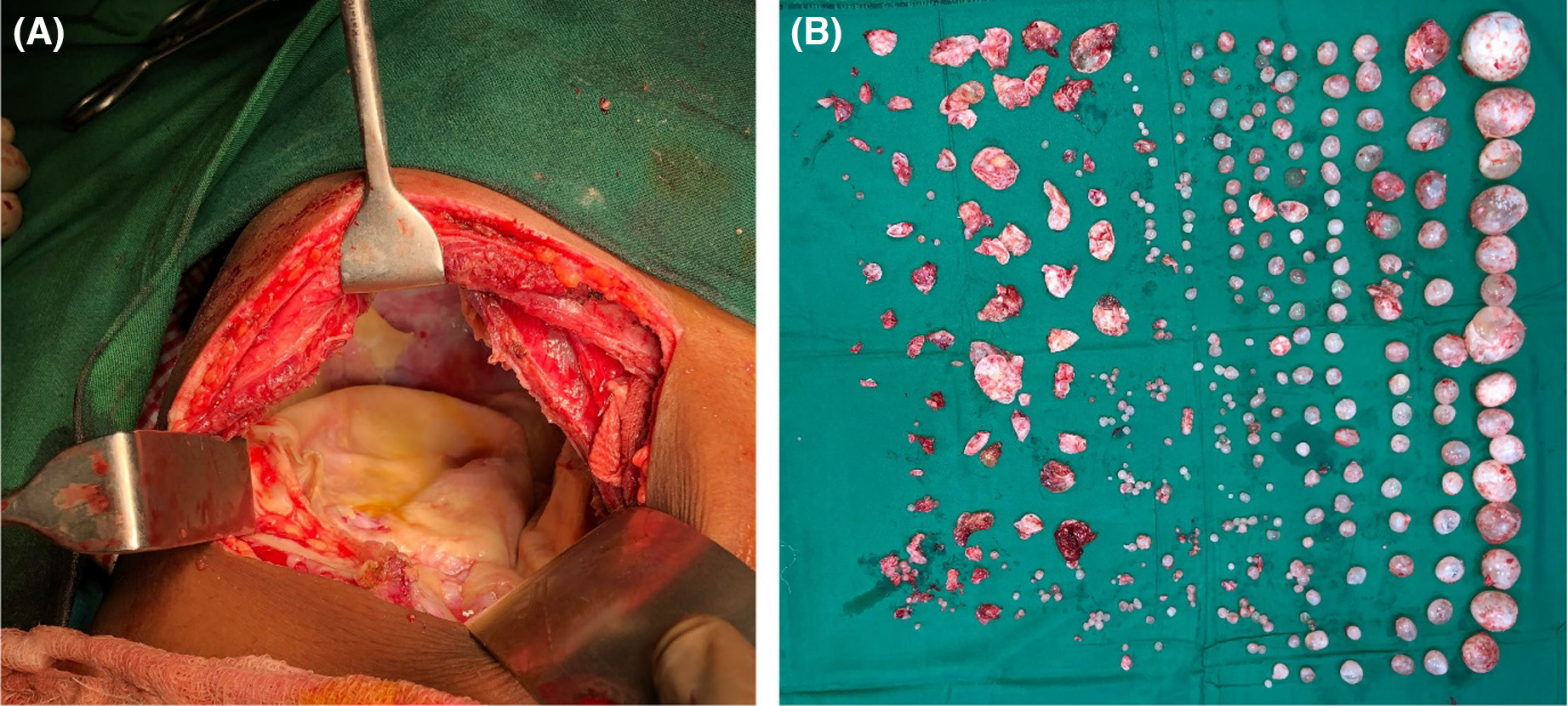
(A) Exploratory laparotomy approach. (B) Extracted hydatid cysts of the patient.

## AUTHOR CONTRIBUTIONS

**Banwari Lal Bairwa:** Conceptualization; data curation; investigation; resources; supervision; validation; visualization; writing – original draft; writing – review and editing. **Ayush Anand:** Conceptualization; data curation; investigation; resources; supervision; validation; visualization; writing – original draft; writing – review and editing.

## FUNDING INFORMATION

The authors did not receive any funding for this manuscript.

## CONFLICT OF INTEREST STATEMENT

The author(s) have no conflict of interests to declare.

## ETHICS STATEMENT

Our institution does not require ethical approval for reporting individual cases or case series.

## CONSENT

Written informed consent was obtained from the patient(s) for their anonymized information to be published in this article.

## GUARANTOR

BLB is the guarantor.

## Data Availability

All data pertaining to this case is available within this manuscript.
